# The Relation between Erythrocyte Trans Fat and Triglyceride, VLDL- and HDL-Cholesterol Concentrations Depends on Polyunsaturated Fat

**DOI:** 10.1371/journal.pone.0047430

**Published:** 2012-10-15

**Authors:** Edmond K. Kabagambe, Jose M. Ordovas, Paul N. Hopkins, Michael Y. Tsai, Donna K. Arnett

**Affiliations:** 1 Department of Epidemiology and the Nutrition Obesity Research Center, School of Public Health, University of Alabama at Birmingham, Birmingham, Alabama, United States of America; 2 Nutrition and Genomics Laboratory, Jean Mayer United States Department of Agriculture Human Nutrition Research Center on Aging, Tufts University, Boston, Massachusetts, United States of America; 3 Department of Internal Medicine, University of Utah, Salt Lake City, Utah, United States of America; 4 Department of Laboratory Medicine and Pathology, University of Minnesota, Minneapolis, Minnesota, United States of America; Brigham & Women’s Hospital, and Harvard Medical School, United States of America

## Abstract

**Background:**

Trans fatty acids (TFA) lower HDL and increase triglyceride concentrations while polyunsaturated fatty acids (PUFA) lower triglycerides and may decrease HDL concentrations. The effect of the interaction between trans fat and PUFA on lipids is uncertain.

**Methods:**

Men and women (n = 1032) in the Genetics of Lipid-Lowering Drugs and Diet Network (GOLDN) study were included. Fatty acids in erythrocyte membranes were measured with gas chromatography while data on potential confounders were obtained from questionnaires. To test the interaction between total erythrocyte PUFA (ePUFA) and TFA (eTFA) on lipid concentrations we distributed eTFA into tertiles and dichotomized ePUFA at the median concentration.

**Results:**

For the 1^st^, 2^nd^ and 3^rd^ tertiles of eTFA, multivariate-adjusted means±s.e.m for HDL were 46.2±1.1, 46.3±1.1 and 45.5±1.0 mg/dL among those with low ePUFA, respectively, while they were 50.0±1.1, 46.9±1.1 and 44.7±1.1 mg/dL among those with high ePUFA, respectively (*P* for interaction = 0.01). For the 1^st^, 2^nd^ and 3^rd^ tertiles of eTFA, multivariate-adjusted means±s.e.m for triglycerides were 178.6±11.3, 144.7±10.9 and 140.8±10.6, respectively, among those with low ePUFA, while they were 133.8±11.3, 145.7±10.9 and 149.3±11.5, respectively, among those with high ePUFA (*P* for interaction = 0.005). Results for VLDL were similar to those for triglycerides. No significant interactions were observed for LDL or total cholesterol.

**Conclusions:**

The relation between trans fat and HDL, VLDL and triglycerides may depend on PUFA. The benefit of avoiding trans fat may be greater among individuals with higher PUFA intake. Supplementation with PUFA among individuals with relatively high trans fat intake may have limited benefits on lipid profiles.

## Introduction

Dietary fat has varying effects on plasma lipoproteins [1]. In general saturated and trans fatty acids increase while monounsaturated fatty acids (MUFA) and omega-6 polyunsaturated fatty acids (PUFA) decrease low-density lipoprotein cholesterol (LDL-C) concentrations [2], [3]. Dietary omega-3 fatty acids are more potent in reducing triglycerides, partly through improved lipoprotein lipase (LPL) activity [4], [5], but have a limited effect on LDL and high-density lipoprotein cholesterol (HDL-C) concentrations [1], [2].

In contrast trans fatty acids, e.g., elaidic acid, up-regulate cholesterol ester transfer protein with concomitant increase in very low-density lipoprotein (VLDL) cholesterol, increase inflammation and down-regulate LPL activity, even in presence of linoleic acid [6], [7], [8]. This may in part explain the decrease in HDL and an increase in triglyceride concentrations following a diet high in trans fatty acids. Although, intake of trans fat has been consistently associated with adverse lipid profiles, a recent study found potential beneficial effects from a ruminant trans fatty acid, vaccenic acid [9]. Even among other non-trans fat subtypes, there are data to suggest differences in their effect on lipoproteins and CHD risk [1], [10], [11], [12].

The metabolic effects of individual dietary fats may also be modified by the overall fatty acid composition of the diet. Indeed fatty acid patterns have been associated with dyslipidemia and the metabolic syndrome in cross-sectional and prospective studies [13], [14]. Most population studies that have examined the relation between dietary fat and lipoproteins have tested for the effects of individual fat subtypes but not their pair-wise interactions. Pair-wise interactions especially between trans fatty acids as a group and total PUFA are of more interest given that PUFA may reduce inflammation and increase LPL activity, thereby improving clearance of triglyceride-rich lipids [4], [5] while trans fatty acids inhibit LPL activity and increase cholesterol ester transfer protein (CETP) activity [7], enzymes with opposing effects on lipids. In this large study we tested whether the association between trans fat in erythrocyte membranes (eTFA), a validated biomarker for dietary trans fat intake, is modified by polyunsaturated fat also measured in erythrocyte membranes (ePUFA).

## Methods

### Ethics Statement

This study was approved by the institutional review boards at the University of Alabama at Birmingham, Tufts University, University of Minnesota and University of Utah. All patients gave written informed consent.

### Study Design and Population

The participants in this study were 1328 white men and women in the Genetics of Lipid Lowering Drugs and Diet Network (GOLDN) family study that enrolled patients from two genetically homogeneous centers in Minneapolis, MN and Salt Lake City, Utah [15]. The GOLDN study is part of the PROgram for GENetic Interaction (PROGENI) Network, a group of National Institutes of Health (NIH)-funded family intervention studies focusing on gene-environment interactions [16]. The main aim of the GOLDN study is to characterize the genetic basis of the variable response of triglycerides to two environmental contexts, one that raises triglycerides (dietary fat), and one that lowers triglycerides (fenofibrate treatment). Men and women in this study participated in a three week open-label clinical trial that tested triglyceride responses to a high-fat milk shake before and after treatment with 160 mg of micronized fenofibrate. After the screening visit (visit 1) and before fenofibrate, the study subjects were asked to suspend use of their lipid-lowering drugs for three weeks and had their lipid profiles and anthropometric measurements taken (visit 2) before an oral fat challenge. The current analysis uses baseline data (visit 2) on anthropometric measurements, fatty acid profiles in erythrocyte membranes, plasma lipid profiles, physical activity and other lifestyle variables to test for the interaction between trans fat and PUFA, both measured in erythrocyte membranes.

### Data Collection

Habitual dietary intake was assessed with a validated National Cancer Institute diet history questionnaire (DHQ) [17], [18], [19] while data on physical activity and other lifestyle variables such as smoking and alcohol intake were collected using an interviewer-administered questionnaire. Fasting glucose, insulin and lipid profiles (e.g., triglycerides, HDL-, LDL-, and total cholesterol) were measured following an eight-hour fast.

### Laboratory Measurements

All samples were centrifuged within 20 minutes of collection at 2000×g for 15 min at 4°C and stored frozen at −70°C. For each analyte, specimens from each participant were assayed in the same batch to eliminate inter-assay imprecision.

Biochemical analyses were performed as previously described [20], [21], [22]. Briefly, triglycerides were measured using a glycerol blanked enzymatic method (Trig/GB, Roche Diagnostics Corporation, Indianapolis, IN) and cholesterol was measured using a cholesterol esterase, cholesterol oxidase reaction (Chol R1, Roche Diagnostics Corporation) on the Roche/Hitachi 911 Automatic Analyzer (Roche Diagnostics Corporation). For HDL-cholesterol, the non-HDL-cholesterol was first precipitated with magnesium/dextran. LDL-cholesterol was measured by a homogeneous direct method (LDL Direct Liquid Select™ Cholesterol Reagent, Equal Diagnostics, Exton, PA). Fasting glucose was measured using the hexokinase-mediated reaction on a Hitachi 911 analyzer (Roche Diagnostics) while fasting insulin was measured using the human insulin specific RIA kit (Linco Research, St. Charles, MO) [23].

Fatty acids in the erythrocyte membranes were extracted with a mixture of chloroform:methanol (2∶1, by volume) collected in heptane and injected onto a capillary Varian CP7420 100 m column using a Hewlett Packard 5890 gas chromatograph equipped with a HP6890A autosampler. The initial temperature of 190°C was increased to 240°C over 50 minutes to separate fatty acids from 12∶0 through 24∶1n9 [24]. A total of 29 fatty acids including 6 trans fatty acids were separated. The performance of the gas chromatography procedure was assessed using 20 blind duplicates in which representative fatty acids were assayed. The CVs were 2.6% for linoleic acid, 2.4% for α-linolenic acid, 2.4% for arachidonic acid, 3.3% for eicosapentaenoic acid, 2.9% docosapentaenoic acid and 2.7% for docosahexaenoic acid [24]. Erythrocyte membrane fatty acids in the GOLDN study have been validated against a diet history questionnaire and reported previously [22]. The reliability coefficients for various assays were excellent and have been reported elsewhere [15].

### Statistical Analysis

SAS Software version 9.2 (SAS Institute, Inc., Cary, NC) was used for statistical analyses. From the 1328 men and women screened for the GOLDN study, we excluded all subjects who did not meet our inclusion criteria [15], those in the top and lowest one percentile of total energy intake and those with missing data on major exposure variables (trans fatty acids and PUFA both in erythrocytes) and potential confounders. The final data set consists of 1032 men and women. The significance of differences in the distribution of categorical variables by the tertiles of erythrocyte trans fat were tested using the chi-square test while continuous variables by ANOVA, if normally distributed, or by the Wilcoxon rank sum test, if not normally distributed.

Lipid profiles, namely HDL, LDL, VLDL, total cholesterol or triglycerides were the outcome variables in mixed multivariate models that simultaneously included all four fat subtypes in erythrocyte membranes, covariates and pedigree as a random effect. The covariates included in the final models were age, sex (men vs. women), BMI, study site, smoking (never, past and current smokers), alcohol intake status (current drinker vs. non-current drinker) and physical activity (as quartiles). Income and education did not change the models appreciably and were excluded from the final analyses. The means and standard errors reported are adjusted for age, sex, BMI, study site, smoking, alcohol intake status, physical activity and pedigree. The differences in the distribution of potential confounders across fatty acid categories were considered significant at *P*≤0.05. The means, standard errors and *P*-values reported for multivariate analyses are from models that included an interaction term for total eTFA and ePUFA.

## Results

### Subject Characteristics

**Table 1** shows the characteristics of the GOLDN study participants by tertiles of trans fat concentrations in erythrocyte membranes and by amount of PUFA also measured in erythrocyte membranes. Among the group with lower PUFA concentrations, individuals in the top tertile of trans fat were significantly (*P*<0.05) younger, had lower waist circumference, blood pressure and fasting glucose when compared to those in the lowest tertile. In the group with higher PUFA concentrations, individuals in the top tertile of trans fat were significantly (*P*<0.05) younger, had lower BMI, waist circumference, were less likely to be alcohol drinkers and had lower fasting glucose compared to those in the lowest tertile. In both the lower and higher groups of ePUFA concentrations, erythrocyte saturated fat concentrations decreased with increase in trans fat intake.

**Table 1:** Characteristics of the study population by polyunsaturated fat and trans fat content, both measured in erythrocyte membranes.

		Low PUFA								High PUFA					
		Low trans		Moderatetrans		High trans		*P*		Low trans		Moderatetrans		High trans	*P*
n	151		171			194	–		193		173		150		–
Age, y	52±14		49±17			45±17	<0.001		54±14		50±15		42±17		<0.0001
BMI, kg/m^2^	29.5±6.2		29.3±6.0		27.9±5.7		0.04		28.9±5.4		27.7±5.0		26.5±5.0		0.0001
Waist circumference, m	1.01±0.18		0.99±0.16		0.95±0.17		0.002		0.98±0.17		0.95±0.17		0.92±0.14		0.01
TV or computer, hr/wkday	2.64±1.85		2.54±1.79		2.72±1.71		0.44		2.78±1.86		2.43±1.78		2.45±1.97		0.03
Women, %	46		50		54		0.34		49		56		57		0.28
Current smokers, %	12		6		8		0.13		7		6		7		0.92
Current drinkers, %	56		49		49		0.27		59		53		42		0.01
Systolic BP, mmHg	119±17		114±17		115±15		0.04		117±18		114±17		114±14		0.18
Diastolic BP, mmHg	70±9		67±10		67±9		0.01		69±9		68±10		67±9		0.15
Insulin, mU/L	14.64±10.00		14.63±8.26		13.70±7.20		0.55		14.23±9.27		12.99±7.13		12.33±6.83		0.44
Glucose, mg/dL	107±24		102±19		98±15		<0.0001		105±22		99±11		98±14		0.0001
HOMA insulin resistance	3.92±2.82		3.80±2.72		3.41±2.10		0.16		3.75±2.75		3.25±2.03		3.08±2.18		0.12
**Erythrocyte fatty acids**															
Total saturated fat, %	35.75±1.41		35.34±1.16		34.58±1.38		<0.0001		35.33±1.03		34.77±1.17		34.18±1.11		<0.0001
Total MUFA, %	18.34±1.02		18.24±1.06		18.42±1.05		0.27		17.55±0.93		17.59±1.01		17.62±0.98		0.81
Total PUFA, %	32.94±1.08		33.14±0.86		32.83±1.14		0.02		35.37±0.85		35.31±0.98		35.19±0.82		0.14
Total cis-n3 fat, %	5.70±0.96		5.41±0.79		5.22±0.84		<0.0001		6.61±1.51		6.21±1.22		5.72±0.91		<0.0001
Total cis-n6 fat, %	27.16±1.33		27.65±1.06		27.54±1.25		0.001		28.69±1.53		29.04±1.44		29.41±1.06		<0.0001
Total trans fat, %	1.23±0.18		1.65±0.12		2.29±0.41		–		1.19±0.19		1.64±0.12		2.15±0.29		–
16∶1 trans, %	0.06±0.02		0.08±0.02		0.10±0.03		–		0.06±0.02		0.07±0.03		0.08±0.03		–
18∶1 trans, %	0.99±0.16		1.35±0.11		1.91±0.37		–		0.98±0.17		1.35±0.12		1.80±0.27		–
18∶2 trans, %	0.18±0.04		0.22±0.04		0.29±0.07		–		0.16±0.04		0.22±0.05		0.27±0.05		–
PUFA:Sat fat ratio	0.92±0.05		0.94±0.04		0.95±0.05		<0.0001		1.00±0.04		1.02±0.05		1.03±0.04		<0.0001
**Dietary data from FFQ**															
Total energy, kcal/d	2062±1010		2063±868		2106±958		0.60		2034±883		2037±765		2068±872		0.86
Total sat fat, % energy	11.78±2.91		11.98±2.69		11.75±2.56		0.54		12.05±3.14		11.70±2.31		12.02±2.44		0.51
Total MUFA, % energy	13.11±3.09		12.95±2.68		12.97±2.56		0.98		14.15±3.11		13.23±2.42		13.40±2.60		0.01
Total PUFA, % energy	7.46±2.26		7.24±1.95		7.32±1.98		0.66		8.33±2.49		7.79±1.95		7.72±2.01		0.06
Total trans, % energy	1.89±0.48		2.08±0.55		2.35±0.64		<0.0001		1.94±0.54		2.15±0.53		2.33±0.69		<0.0001
Carbohydrate, % energy	47.86±9.34		50.27±8.31		51.45±7.24		0.0004		45.45±9.67		49.16±7.08		49.54±6.70		0.0002
Protein, % energy	15.97±3.04		15.53±2.80		15.25±2.77		0.11		16.23±3.03		16.23±2.70		15.86±2.36		0.36

Values are means±SD or %. PUFA = Polyunsaturated fat; MUFA = Monounsaturated fat; Sat = Saturated; Trans = Total trans fat in erythrocyte membranes; BP = Blood pressure; FFQ = Food frequency questionnaire.

In the total study sample, the mean (±SD) erythrocyte membrane trans fat content as a percentage of total erythrocyte membrane fat was 1.21±0.19, 1.64±0.12 and 2.23±0.37 for the 1^st^, 2^nd^ and 3^rd^ tertile of total trans fat, respectively. Thus, individuals in the top tertile had 84% higher trans fat on average compared to those in the lowest tertile. The amount of energy from trans fat estimated from a food frequency questionnaire was also higher in top tertile compared to the lowest tertile of erythrocyte trans fat in both the low and high erythrocyte membrane PUFA groups (Table 1). As expected, the percentage of energy intake from PUFA was also higher in the higher compared to lower erythrocyte membrane PUFA group (Table 1).

In fully adjusted models that included main effects and an interaction term for eTFA and ePUFA, erythrocyte total trans fat concentrations showed a significant inverse association with HDL (*P = *0.01) but no association was observed for LDL (*P = *0.46), triglycerides (*P = *0.79) or VLDL (*P = *0.51). In the same model, ePUFA showed a significant inverse association with triglycerides (*P = *0.01) and VLDL (*P = *0.004) but no association was observed between PUFA and LDL (*P = *0.91) or HDL (*P = *0.15).

**Figure 1:**
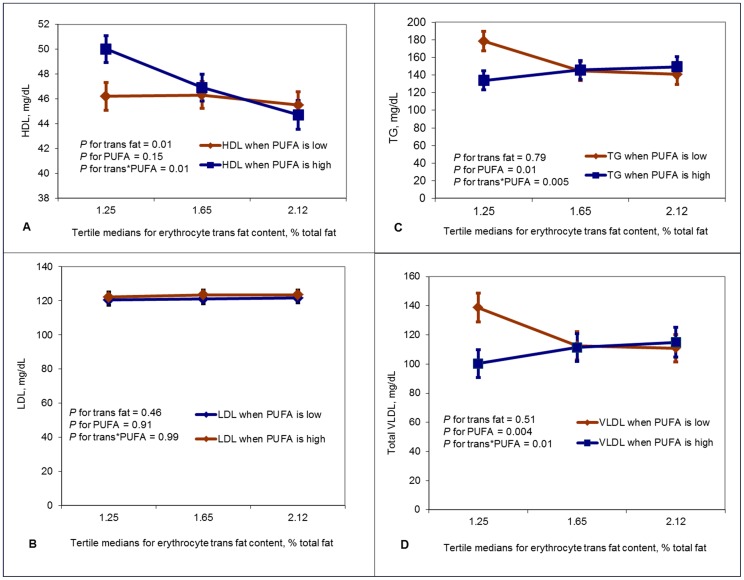
Relation between trans fat (distributed in tertiles) and HDL (panel A), LDL (panel B), triglycerides (panel C) and VLDL (panel D) in analyses stratified by polyunsaturated fat content in erythrocyte membranes. Lipid values are means ± s.e.m and are adjusted for study site, age, sex, body mass index, physical activity, alcohol intake status, smoking status, erythrocyte monounsaturated fat, erythrocyte saturated fat and pedigree as a random effect.

**Figure 1** shows multivariate-adjusted associations between eTFA, ePUFA and their interactions in relation to fasting lipid concentrations. For the 1^st^, 2^nd^ and 3^rd^ tertiles of trans fat, multivariate-adjusted means ± s.e.m for HDL were 46.2±1.1, 46.3±1.1 and 45.5±1.0 mg/dL among those with low ePUFA concentrations, respectively, while they were 50.0±1.1, 46.9±1.1 and 44.7±1.1 mg/dL among those with high ePUFA concentrations, respectively (*P* for interaction = 0.01) **(** **Figure 1** **panel A)**.

There was no significant interaction (*P* for interaction = 0.99) between eTFA, ePUFA and LDL cholesterol concentrations **(** **Figure 1** **panel B).**

The relation between eTFA, ePUFA and triglycerides was similar to that of VLDL **(** **Figure 1** **panels C and D).** For the 1^st^, 2^nd^ and 3^rd^ tertiles of eTFA, multivariate-adjusted means ± s.e.m for triglycerides were 178.6±11.3, 144.7±10.9 and 140.8±10.6, respectively, among those with low ePUFA, while they were 133.8±11.3, 145.7±10.9 and 149.3±11.5, respectively, among those with high ePUFA concentrations, (*P* for interaction = 0.005) **(** **Figure 1** **panel C)**. As for triglycerides, we observed a significant inverse association (*P* = 0.004) between ePUFA and VLDL, no main effect for eTFA (*P* = 0.51) and a significant interaction between eTFA and ePUFA (*P* = 0.01) **(** **Figure 1** **panel D)**. A non-significant inverse association between eTFA and VLDL was observed among individuals in the low ePUFA group i.e., multivariate-adjusted VLDL means ± s.e.m of 138.7±9.9, 112.5±9.6 and 110.9±9.3 for the low, moderate and high eTFA concentrations, respectively. A non-significant positive association between eTFA and VLDL was observed among individuals in the high ePUFA group i.e., 100.3±9.9, 111.4±9.6 and 115.0±10.1, for the low, moderate and high eTFA concentrations, respectively. No significant eTFA or ePUFA main effects or interactions were observed for total cholesterol concentrations (**Table S1)**.

Since saturated fat and MUFA in erythrocytes are not good biomarkers of intake, we performed additional analyses using saturated fat and MUFA variables from the FFQ as covariates. We also performed analyses adjusting for carbohydrate intake. These analyses did not change the inferences from models that were not adjusted for these variables.

## Discussion

Consistent with other studies, we found that higher concentrations of trans fatty acids in erythrocyte membranes are associated with lower HDL concentrations and higher PUFA concentrations are associated with lower triglyceride and VLDL concentrations [3], [25], [26]. However, the relation between erythrocyte trans fat and HDL varied with the level of erythrocyte PUFA; this observation has not been reported before. Similarly, the relation between erythrocyte PUFA and triglyceride or VLDL concentrations varied depending on the amount of erythrocyte trans fat suggesting that the expected benefit of PUFA on TG or VLDL is abrogated at moderate to high intakes of trans fat. It is possible that the benefit of avoiding trans fat is greater among individuals with higher PUFA intake. Supplementation with PUFA among individuals with relatively high trans fat intake may have a limited benefit on lipid profiles.

Compared to other studies on the relation between fatty acids and lipids, our study had a number of strengths including the large sample size (n = 1032) and use of objectively measured independent (erythrocyte fatty acids) and dependent variables (lipids). Use of erythrocyte membrane fatty acids measured by gas chromatography greatly enhances the quality of trans fat and PUFA assessments. Furthermore, our analyses adjusted for various potential confounders including age, sex, study site, body mass index, physical activity, alcohol intake, smoking, erythrocyte monounsaturated fat, erythrocyte saturated fat and pedigree as a random effect.

When the erythrocyte PUFA concentration is above the median in our study population, we see the expected associations [26], [27], i.e., an inverse association between erythrocyte trans fat and HDL (Figure 1 panel A) and a positive association between trans fat and triglycerides and VLDL (Figure 1 panels C and D). However, when erythrocyte PUFA is low there is no relation between trans fat and HDL; and triglyceride and VLDL concentrations even tend to decrease at higher trans fat concentrations in the low PUFA group. Also, at low trans fat concentrations individuals with low erythrocyte PUFA concentrations have higher VLDL and triglyceride concentrations compared to those with higher concentrations of PUFA.

The exact reasons for these observations are not clear but could be related to trans fat inhibition of LPL activity and up-regulation of CETP activity [7] and/or up-regulation of LPL activity by PUFA [4]. It is possible that at low trans fat concentrations, CETP activity is low favoring HDL increase but in individuals with low PUFA concentrations there is insufficient LPL activity. Less LPL activity would result in delayed lipolysis of triglyceride-rich lipoproteins with resultant accumulation of VLDL and triglycerides. Indeed, individuals with higher erythrocyte PUFA had lower VLDL and triglyceride concentrations as expected. The low HDL concentrations observed at high trans fat concentrations in both the low and high PUFA groups could mean that the acute elevation in CETP activity (known to lower HDL) by higher amounts of trans fat [7] cannot be compensated by an increase in LPL activity due to higher PUFA concentrations. Thus, the trans fat effect may dominate the PUFA effect resulting in low HDL. Furthermore, trans fatty acids are associated with higher concentrations of biomarkers of inflammation [8] and proinflammatory cytokines are known to down-regulate LPL activity [28]. This may partly explain why the known benefit of PUFA on triglycerides is not evident at higher concentrations of trans fat. The clinical implication of the current observations is that response to fish oil or lipid-lowering medications based on PUFA esters may be lower in patients with higher trans fat intake.

Our study has a number of limitations. It was a cross-sectional design thus we are unable to determine whether the observed associations may have been affected by reverse causality since individuals with dyslipidemia may have changed their diet. Secondly, we did not measure LPL or CETP activities so as to better understand the underlying mechanisms. Nonetheless, these findings are interesting in that they show for the first time that the effect of trans fat or PUFA vary depending on relative concentrations of other fatty acids. Our study is unique in that lipids were measured three weeks after patients suspended use of their lipid-lowering drugs. Thus our findings are not confounded by lipid-lowering drugs.

These findings will need to be replicated in prospective studies with lipids and/or cardiovascular events as end-points. Other human or animal studies are needed to elucidate on the mechanism underlying the observed interaction between trans fat, PUFA and lipids (i.e., HDL, triglycerides and VLDL).

### Conclusion

The association between trans fat and lipids (HDL, VLDL and triglycerides) may vary depending on PUFA. The benefit of avoiding trans fat may be greater among individuals with higher PUFA intake. Supplementation with PUFA among individuals with relatively high trans fat intake may have limited benefits on lipid profiles.

## Supporting Information

Table S1 **Interaction between erythrocyte trans fat and polyunsaturated fat on HDL, LDL, total cholesterol and triglyceride concentrations.**
(DOC)
