# Supplementary material for: The Relation between Erythrocyte Trans Fat and Triglyceride, VLDL- and HDL-Cholesterol Concentrations Depends on Polyunsaturated Fat
Source: PLoS One. 2012 Oct 15;7(10):e47430. doi: 10.1371/journal.pone.0047430 (PMC3471823; doi:10.1371/journal.pone.0047430)
Supplement: Table S1 — Interaction between erythrocyte trans fat and polyunsaturated fat on HDL, LDL, total cholesterol and triglyceride concentrations. (DOC) [file pone.0047430.s001.doc]

The relation between erythrocyte trans fat and triglyceride, VLDL- and HDL-cholesterol concentrations depends on polyunsaturated fat

Edmond K Kabagambe,^1*^ Jose M. Ordovas,^2^ Paul N. Hopkins,^3^ Michael Y. Tsai^4^ and Donna K. Arnett^1^

1 Department of Epidemiology and the Nutrition Obesity Research Center, University of Alabama at Birmingham, School of Public Health, Birmingham, Alabama, United States of America,

2 Nutrition and Genomics Laboratory, Jean Meyer United States Department of Agriculture Human Nutrition Research Center on Aging, Tufts University, Boston, Massachusetts, United States of America,

3 Department of Internal Medicine, University of Utah, Salt Lake City, Utah, United States of America,

4 Department of Laboratory Medicine and Pathology, University of Minnesota, Minneapolis, Minnesota, United States of America.

* E-mail: [edmond.kabagambe@vanderbilt.edu](mailto:edmond.kabagambe@vanderbilt.edu)

**Table S1.** Interaction between erythrocyte trans fat and polyunsaturated fat on HDL, LDL, total cholesterol and triglyceride concentrations.

|  |  |  | Tertiles of erythrocyte membrane trans fat^1^ | | | |  | *P* | | | | |
| --- | --- | --- | --- | --- | --- | --- | --- | --- | --- | --- | --- | --- |
|  | Erythrocyte PUFA |  | Low trans |  | Moderate trans | High trans |  | Trans |  | PUFA |  | Trans*PUFA |
| Erythrocyte membrane trans fat content, % | -- |  | 1.21± 0.19 |  | 1.64± 0.12 | 2.23 ± 0.37 |  | -- |  | -- |  | -- |
|  |  |  |  |  |  |  |  |  |  |  |  |  |
| HDL-cholesterol, mg/dL | Low |  | 46.2 ± 1.1 |  | 46.3 ± 1.1 | 45.5 ± 1.1 |  | **0.01** |  | 0.15 |  | **0.01** |
|  | High |  | 50.0 ± 1.1 |  | 46.9 ± 1.1 | 44.7 ± 1.1 |  |  |  |  |  |  |
|  |  |  |  |  |  |  |  |  |  |  |  |  |
| Triglycerides, mg/dL | Low |  | 178.6 ± 11.3 |  | 144.7 ± 10.9 | 140.8 ± 10.6 |  | 0.79 |  | **0.01** |  | **0.005** |
|  | High |  | 133.8 ± 10.8 |  | 145.7 ± 10.9 | 149.3 ± 11.5 |  |  |  |  |  |  |
|  |  |  |  |  |  |  |  |  |  |  |  |  |
| Total VLDL, mg/dL | Low |  | 138.7 ± 9.9 |  | 112.5 ± 9.6 | 110.9 ± 9.3 |  | 0.51 |  | **0.004** |  | **0.001** |
|  | High |  | 100.3 ± 9.5 |  | 111.4 ± 9.6 | 115.0 ± 10.1 |  |  |  |  |  |  |
|  |  |  |  |  |  |  |  |  |  |  |  |  |
| Total cholesterol, mg/dL | Low |  | 192.8 ± 3.5 |  | 190.4 ± 3.4 | 189.3 ± 3.2 |  | 0.34 |  | 0.48 |  | 0.95 |
|  | High |  | 193.8 ± 3.3 |  | 193.5 ± 3.3 | 191.5 ± 3.5 |  |  |  |  |  |  |
|  |  |  |  |  |  |  |  |  |  |  |  |  |
| LDL-cholesterol, mg/dL | Low |  | 120.4 ± 2.9 |  | 121.0 ± 2.8 | 121.6 ± 2.7 |  | 0.46 |  | 0.91 |  | 0.99 |
|  | High |  | 122.2 ± 2.8 |  | 123.4 ± 2.8 | 123.4 ± 3.0 |  |  |  |  |  |  |

Values are means ± SD for trans fat content or means ± s.e.m for lipid concentrations. PUFA = Polyunsaturated fat; Trans = Trans fat

^1^ Lipid values are adjusted for study site, age, sex, body mass index, physical activity, alcohol intake status, smoking status, erythrocyte monounsaturated fat, erythrocyte saturated fat and pedigree as a random effect.
